# Relationship between working memory performance and neural activation measured using near-infrared spectroscopy

**DOI:** 10.1002/brb3.238

**Published:** 2014-05-24

**Authors:** Yutaro Ogawa, Kiyoshi Kotani, Yasuhiko Jimbo

**Affiliations:** Graduate School of Frontier Science, The University of TokyoChiba, Japan

**Keywords:** Near-infrared spectroscopy, working memory, working memory performance

## Abstract

**Background:**

Working memory (WM) is a key function for various cognitive processes. Near-infrared spectroscopy (NIRS) is a powerful technique for noninvasive functional imaging. However, a study has yet to be published on the application of NIRS for evaluating WM performance. The objective was to evaluate NIRS for measuring WM performance.

**Methods:**

Subjects were trained to perform a visuospatial WM task. Eight channels on the lateral prefrontal cortex were analyzed. We asked the following three questions: (1) Does WM performance correlate with NIRS signal amplitudes? (2) What are the differences in NIRS amplitudes between correct- and incorrect-WM tasks? (3) Is there a correlation between WM performance and NIRS amplitudes in only correct-WM tasks?

**Results:**

NIRS activation in all channels correlated with WM performance (*P* < 0.05). There was a statistically significant difference (*P* < 0.05) in seven channels between NIRS amplitude in correct- and incorrect-WM tasks. NIRS activation of the delay time averaged with only correct-WM tasks, correlated with WM performance in six channels (*P* < 0.05).

**Conclusions:**

Subjects with better WM performance have higher levels of oxyhemoglobin activation compared with control trials in the WM delay time, and our results suggest that NIRS will be useful for measuring the WM performance.

## Introduction

Working memory (WM) refers to the process of actively maintaining relevant information for brief periods of time (Baddeley 1992a,b; Bear et al. 2001). In a typical WM test, a sample stimulus is presented to subjects, followed by a delay of several seconds, and then a test stimulus is presented. The subjects answer whether the test stimulus matches the sample (Pessoa et al. 2002). WM is a key requirement for various cognitive processes, such as planning and reasoning (Baddeley 1992a), and these processes are important for intellectual work. Furthermore, WM performance is higher in healthy individuals compared with patients with certain psychiatric disorders, such as schizophrenia (Conklin et al. 2000; Twamley et al. 2006), depression (Suto et al. 2004), and bipolar disorder (Kameyama et al. 2006).

Currently, behavioral performance and neural activities during WM tasks are the subject of investigations on the neural mechanism of WM (Pessoa et al. 2002; D'Esposito 2007). Measuring the neural activities has advanced compared with measuring only behavioral performance. For example, neural activities during verbal fluency tasks are measured to aid the diagnosis of psychiatric disorders (Suto et al. 2004; Kameyama et al. 2006; Womelsdorf et al. 2007; Cyranoski 2011) because some psychiatric disorders are difficult to distinguish from behaviors alone. Neural mechanisms of WM have been extensively investigated in monkeys. Funahashi et al. (1989) demonstrated that neurons in the prefrontal cortex emit stimulus-specific sustained discharge during the delay period in the WM paradigm. In humans, functional magnetic resonance imaging (fMRI) studies, which measure the hemodynamic change resulting from neuronal activation, support the role of prefrontal regions in WM (McCarthy et al. 1994, 1996; D'Esposito 2007).

Understanding the relationship between neuronal activities and WM performance is important to assist the diagnosis of certain psychiatric disorders and measure intellectual fatigue that occurs in various cognitive processes. In an fMRI study, Pessoa et al. (2002) showed that the blood oxygenation level dependency (BOLD) amplitude in the delay period predicts WM performance.

Near-infrared spectroscopy (NIRS) is a technique for noninvasive functional imaging (Huppert et al. 2009; Hoshi et al. 2011; Machado et al. 2011; Tsytsarev et al. 2012). It detects changes in regional cerebral blood volume by measuring the levels of oxygenated- and deoxygenated-hemoglobin (oxy-Hb and deoxy-Hb, respectively) (Strangman et al. 2002; Yamanaka et al. 2010). Although fMRI also measures hemodynamic changes in the brain, NIRS has certain advantages, such as compactness and low restriction. NIRS is approved as an “advanced medical technology” to assist psychiatric diagnoses in Japan (Cyranoski 2011). Previous research using NIRS demonstrated increasing oxy-Hb levels in the lateral prefrontal cortex (LPFC) during WM (Tsujimoto et al. 2004; Aoki et al. 2011; Sato et al. 2011). However, to the best of our knowledge, no published study addresses the correlation between NIRS signals and WM performance.

In the present study, we investigated the relationship between hemodynamic changes measured using NIRS while performing a visuospatial WM task. We analyzed the correlation between NIRS amplitudes and WM performance for all subjects and compared the difference in oxy-Hb levels between correct-WM and incorrect-WM tasks. Moreover, we analyzed the correlation between NIRS amplitudes in only correct-WM tasks and WM performance for all subjects. The results of these studies indicated that NIRS will be useful for measuring WM performance.

## Methods

### Experimental procedures

**Figure 1:**
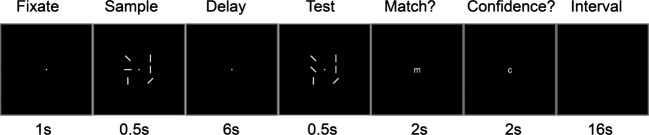
Experimental design of the visuospatial working memory (WM) task (one trial). The subjects answered whether the sample stimuli matched the test stimuli by pressing the button under “Match?” for 2 sec. After pressing “Match?,” subjects also indicated their confidence by pressing the button under “Confidence” for 2 sec. Each set comprised 20 trials, including five control tasks (easy task with four bars) and 15 test tasks (with six bars). Each subject performed four sets. This experimental design is a slightly modified version of the WM task described in an fMRI study (Pessoa et al. 2002).

Subjects were trained to perform a visuospatial WM task (Yamanaka et al. 2010) (Fig. 1). Each trial lasted for 28 sec. Each subject performed four blocks of sets, and each block comprised 20 WM trials (Fig. 1). The orders of sets were counterbalanced among subjects. WM trials comprised fixation (1 sec), a sample visual display (0.5 sec), a delay period (6 sec), a test visual display (0.5 sec), two response periods (2 sec each), and a final intertrial interval (16 sec). Each sample and test visual displays comprised a fixation spot and an array of six oriented white bars (vertical, horizontal, 45°, or −45°, respectively). During the “match” response period, subjects pressed a button with their right index finger (“match”) or right middle finger (“non-match”). “Match” meant the test display was the same as the sample test and “non-match” meant a change in the orientation of a single bar. In the “confidence” response period, subjects pressed a button with their right index finger (“high confidence”) or right middle finger (“low confidence”).

In each set, one-half of the WM trials involved a change in the test display. In each set comprising 20 trials, five trials of the 20 were easy controls in which the sample and test displays consisted of a fixation spot and an array of only four oriented white bars.

A “correct-WM task” was defined as a “match or non-match” response when the answer was correct and the confidence was high. Therefore, we attempted to minimize the contributions of guesses. “Incorrect-WM tasks” included other responses. Control tasks were defined in the same manner. “WM performance” was defined as the ratio of the correct-WM tasks to the incorrect-WM tasks.

This experimental design was a slightly modified version used by Pessoa et al. (2002) in fMRI studies. We modified three variables. First was the difficulty of the task, which involved eight bars in the original experiment. However, the eight bar task was too difficult, and there were only a few “high-confidence” answers. Therefore, we limited the number of bars to six. Second was the length of the time interval. It was modified from 2 to 16 sec in the present study because NIRS measurements require a longer sampling interval. Third was the number of control tasks, which we changed from zero to four bars.

We recruited 11 healthy men, with a mean age of 23.1 ± 1.2 (mean ± standard deviation) years. All were right-handed with normal or corrected-to-normal vision. This study was approved by the Ethics Committee of the Graduate School of Frontier Sciences (The University of Tokyo). All subjects gave their informed consent after being given a full written description of the study.

### Signal processing

**Figure 2:**
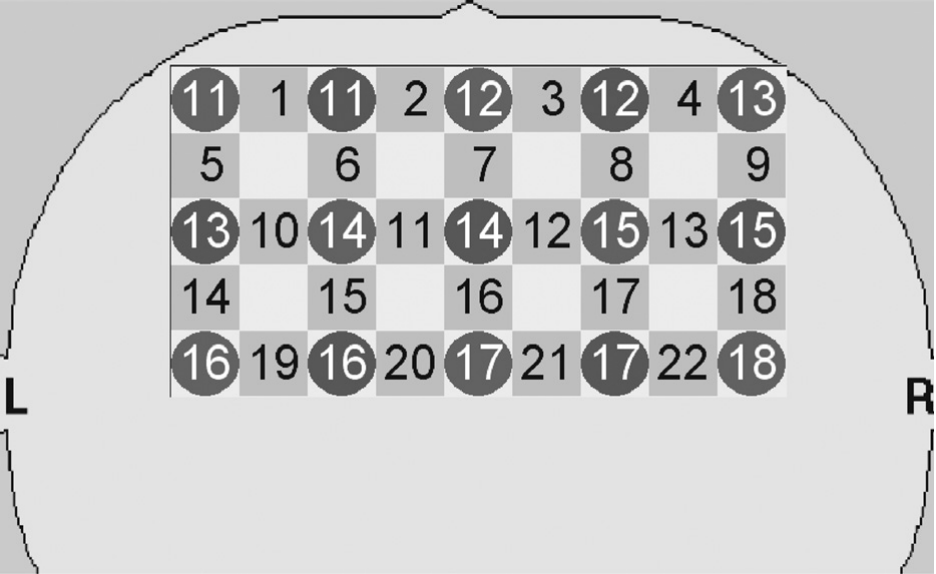
Near-infrared spectroscopy (NIRS) probes (circles) and measurement channels (squares). Signals generated by the lateral prefrontal cortex were detected in channels 10, 13–15, 17–19, and 22.

NIRS data were acquired using a 24-channel NIRS system (ETG-4000, Hitachi Medical Corporation, Tokyo, Japan). Changes in Hb levels were measured using 695 and 830 nm near-infrared light to detect oxy-Hb and deoxy-Hb, respectively. NIRS measured the hemoglobin at the surface of the cerebral cortex at a depth of 20–30 mm from the surface of the scalp (Hock et al. 1997; Cui et al. 2011). Figure 2 illustrates the channel locations. NIRS probes were fixed with thermoplastic 3 × 5 shells (frontal lobe: 22 channels) with the lowest frontal probes positioned along the Fp1-Fp2 line according to the international 10–20 system. The absorption of near-infrared light was measured at a sampling rate of 10 Hz.

In the present study, we focused on oxy-Hb under the assumption that oxy-Hb levels reflect cognitive activation more directly and correlate more strongly with BOLD signals compared with fMRI measurements of deoxy-Hb levels (Cui et al. 2011). Calculations of changes in oxy-Hb levels were performed using a software provided with the ETG-4000, which is based on the “modified Beer–Lambert law” (Maki et al. 1995). To analyze the oxy-Hb data, the moving average method (moving average window: 5 sec) was used to remove high-frequency noise. Then, the activation period was defined as 24 sec (Task [12 sec] + Relax [12 sec]) from the start of the task. The pre- and postactivation baseline periods were defined as 4 sec before and after the activation period. The coefficient of linear regression was determined from these two baseline periods using the least square method. A first order detrend was applied to the change in oxy-Hb levels during the activation period to remove low-frequency noise. Then, the averaged change in oxy-Hb levels during 5–7 periods after starting the task was expressed as “the activation of the sample stimulus,” and the averaged change during 7–11 periods was expressed as “the activation of the delay time.” In NIRS analyses, hemodynamic reaction involved several time delays, and these occurred with the same timing as previously reported in fMRI studies study by Pessoa et al. (2002).

### Data analysis

#### Correlation between WM performance and NIRS signals

To analyze the relationship between changes in oxy-Hb levels and WM performance, the activations of the delay time were averaged for all trials (60 trials for tasks and 20 trials for controls). The activation index *x*(*i*,ch) was calculated for each subject to exclude individual differences as follows:


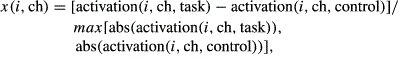
(1)

where *i* means the subject, ch means the analyzed channel, and “abs” is the absolute value. This calculation is the same as that described in the fMRI study by Pessoa et al. (2002).

To assess the correlation between WM performance and change in oxy-Hb levels, we employed a logistic regression analysis according to the previous study about WM performance and fMRI signals (Pessoa et al. 2002). We fit the data in generalized linear models with the logit link function. The logit link function means


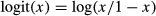
(2)

The response variable *y*(*i*) was the subjects' task accuracy following the binomial distribution. The predictor variable was the activation index *x*(*i,*ch) of the delay time averaged for all tasks (60 trials per subject) of each channels. Thus, we modeled as



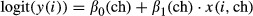
(3)

The logistic regression analysis was carried out in each channel. In this study, the analyzed channels were 10, 13–15, 17–19, and 22, because WM involves the function of the LPFC (Tsujimoto et al. 2004). Then, we tested the statistical significance of the predictive effects of NIRS signals for WM performance (whether the values of *β*_1_(ch) were reliably greater than zero) by the likelihood ratio test. We compared the likelihoods of the mode (3) with those of the model:



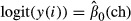
(4)

This statistic followed asymptotically a chi-square distribution with 1 degree of freedom. The *P*-values were obtained for each channel and we corrected the multiple comparisons according to the false discovery rate (FDR) of 0.05.

#### Difference between correct- and incorrect-WM tasks

We next analyzed the difference in oxy-Hb levels between correct- and incorrect-WM task responses. The activations induced by the sample stimulus and those of the delay time were averaged for each response. Using a paired *t*-test (Wilcoxon signed-rank test) to analyze the data for all subjects and a FDR of 0.05, we determined whether there were statistically significant differences between correct- and incorrect-WM task responses in the eight channels for the LPFC.

#### Correlation between WM performance and NIRS signals specific to correct-WM task response

There are two hypotheses as the mechanism that the subject with better WM performance has higher levels of NIRS activation index. The first argues that oxy-Hb levels in incorrect-WM tasks are lower than that of correct-WM tasks. The second hypothesis maintains that oxy-Hb levels for the lower performers are lower than that of the higher performers even for correct-WM tasks. To investigate the second hypothesis, the activation index for correct-WM task was calculated for each subject and the logistic regression analysis was carried out in the manner described above.

## Results

### Task behavior

The mean and standard deviation for the WM performance (task accuracy) in six-bar task trials for all subjects was 0.73 ± 0.12. According to this result, we analyzed the NIRS signals of 10 subjects. Performance of one subject was excluded from this analysis because it was judged as too high (only seven incorrect tasks). Therefore, an average of approximately 10 tasks was set as a benchmark to analyze NIRS signals. There was no statistically significant change in WM performance during four sets according to the results of paired *t*-test. Mean performance in four bar control trials was 0.95 ± 0.1 for all subjects.

### Correlation between WM performance and NIRS signals

**Figure 3:**
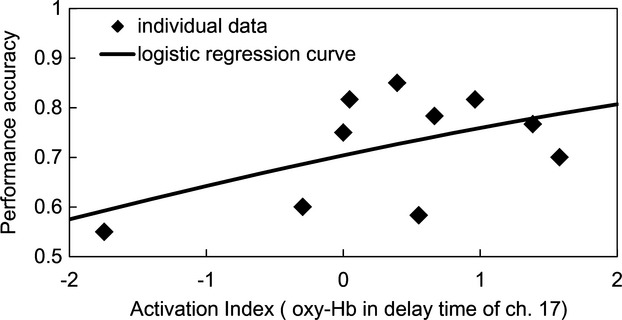
The correlation between near-infrared spectroscopy (NIRS) activation index of oxygenated hemoglobin (oxy-Hb) and working memory (WM) performance as determined from the delay time in channel 17. Activation indices of the delay time in all eight channels correlated with WM performance (*P* < 0.05).

To assess the correlation between WM performance and change in oxy-Hb levels, we employed a logistic regression analysis. The analyses were performed for each channel (10, 13–15, 17–19, and 22) for the LPFC. Figure 3 shows the results of the delay time for channel 17. The odds ratio was 1.33 and its Wald confidence interval (CI) were from 1.09 to 1.61. The lower values of CI were larger than 1.0 in all eight channels. The goodness-of-fit index (GFI) of channel 17 was 0.22. The maximum value of the GFI was 0.53 of channel 10. The predictive effects of NIRS signals for WM performance were tested for significance by the likelihood ratio test. The coefficients of activation index were reliably greater than zero (*P* < 0.05) for all eight channels. Multiple comparisons were corrected according to the FDR (0.05). These results indicate that better WM performance correlated with elevated oxy-Hb levels (delay times) in the LPFC compared with controls.

### Difference between correct- and incorrect-WM tasks

**Figure 4:**
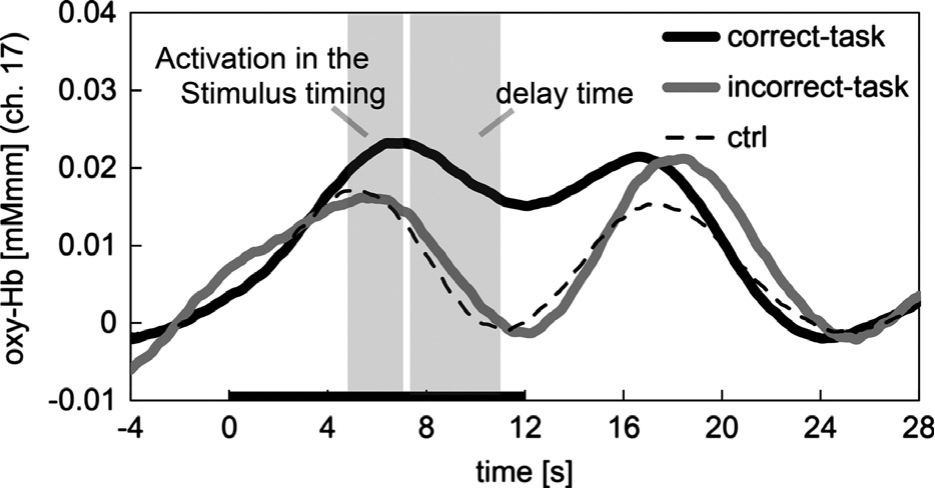
Oxygenated hemoglobin (oxy-Hb) time-series data for correct-working memory (WM) task, incorrect-WM task, and control measured in channel 17 (average of all subjects). The left shaded column (5–7 sec) indicates the stimulus timing, the right shaded column (7–11 sec) indicates the delay time, and the black bar on the *x*-axis indicates the task period.

**Figure 5:**
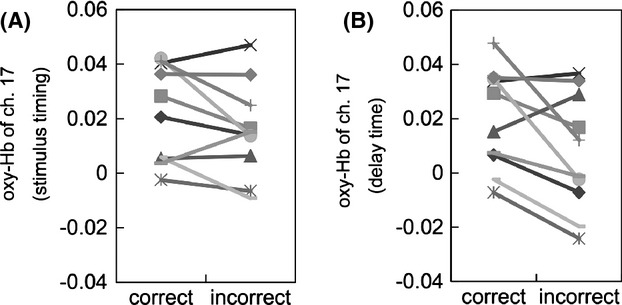
The difference in oxygenated hemoglobin (oxy-Hb) levels, measured in channel 17, between correct- and incorrect-working memory (WM) task in stimulus timing and delay time analyzed using a paired *t*-test. (A) There was no statistical significant difference in the stimulus timing after the correction of multiple comparisons. (B) There were statistically significant differences (*P* < 0.05) in the delay time measured in seven channels (10, 13, 14, 17–19, and 22) after the correction of multiple comparisons.

Differences between oxy-Hb levels in correct- and incorrect-WM tasks were analyzed. Figure 4 shows the oxy-Hb time-series data of channel 17, averaged among subjects. Black and gray lines indicate the change for the correct- and incorrect-WM tasks, respectively, and the dotted line indicates the control task. There were no statistically significant differences (*P* < 0.05) in the stimulus timing for all channels after the correction of multiple comparisons. In contrast, there were statistically significant differences (*P* < 0.05) in seven channels (10, 13, 14, 17–19, and 22) after the correction of multiple comparisons (Fig. 5). These results indicate that changes in oxy-Hb levels detected for correct-WM were higher than those of incorrect-WM task in delay time.

### Correlation between WM performance and NIRS signals specific to correct-WM task response

**Figure 6:**
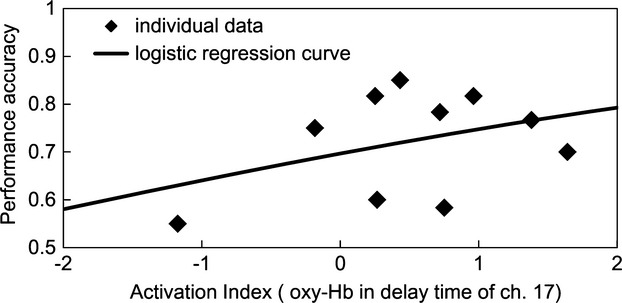
The correlation between near-infrared spectroscopy (NIRS) activation index in the delay time of channel 17 calculated for only correct-working memory (WM) task and WM performance. Activation indices of the delay time in six channels (10, 14–17, 19, and 22) correlated significantly with WM performance (*P* < 0.05).

The correlation between the activation index of the delay time averaged for only correct-WM tasks and WM performance was evaluated using logistic regression analysis. The results for channel 17 indicated that oxy-Hb levels for subjects with better WM performance was higher for correct-WM trials compared with control trials in delay time (Fig. 6). The odds ratio was 1.29 and its CI were from 1.02 to 1.62. The lower values of CI were larger than 1.0 in six channels (10, 14–17, 19, and 22). The GFI of channel 17 was 0.12. The maximum value of the GFI was 0.34 of channel 10. The predictive effects of NIRS signals for WM performance were tested for significance by the likelihood ratio test. The activation indices of the delay times in six channels (10, 14–17, 19, and 22) correlated with WM performance (*P* < 0.05). Multiple comparisons were corrected according to the FDR (0.05).

## Discussion

The objective of the present study was to determine whether NIRS could measure WM performance. We first demonstrated a significant correlation between WM performance and oxy-Hb levels with respect to the average delay time and then determined whether this correlation corresponded to the activation indices of eight channels (10, 13–15, 17–19, and 22) (Fig. 3). Moreover, subjects with better WM performance generated higher oxy-Hb levels in the LPFC compared with control trials.

There are two hypotheses to explain the mechanism that governs the correlation between the WM performance and NIRS data. The first argues that oxy-Hb levels in incorrect-WM tasks are lower than that of correct-WM tasks. Because low performers answer incorrectly more often, oxy-Hb levels are lower compared with high performers. The second hypothesis maintains that oxy-Hb levels for the lower performers are lower than that of the higher performers even for correct-WM tasks. Our analyses were designed to confirm the accuracy of the valid hypothesis.

Therefore, we analyzed the differences in oxy-Hb levels between correct- and incorrect-WM task performances. With respect to the delay time, there were statistical significant differences in seven channels (10, 13, 14, 17–19, and 22) that monitored the LPFC, indicating that oxy-Hb levels were higher for correct-WM task compared with incorrect-WM task performance. These results are consistent with those of fMRI studies (D'Esposito 2007), and higher oxy-Hb levels may have been caused by the sustained discharge of neurons that are essential for WM (Funahashi et al. 1989).

Our results showed that the timing of the stimulus that induced oxy-Hb activations did not have statistical significant difference between correct- and incorrect-WM tasks. In contrast, there was a statistically significant difference in the delay time for this response. Therefore, we conclude that task failure correlated with the delay time (WM), but not in stimulus timing, thereby supporting the first hypothesis that the oxy-Hb levels in the incorrect-WM task are lower than that in correct-WM task.

We analyzed the correlation between WM performance and the activation index averaged for only correct-WM tasks, and we observed that the activation indices of the delay time in six channels correlated with WM performance (*P* < 0.05). This result indicated that oxy-Hb levels for correct-WM task of the poor performers were lower than those of the high performers. Although our experiments do not define the mechanism(s) responsible for these findings, we believe that it is reasonable to conclude that an individual's WM capacity accounts for the difference. Therefore, a person with higher WM capability may generate higher NIRS signals compared with control and task trials. In a previous study, using electroencephalogram (EEG)/magnetoencephalogram (MEG) study, Palva et al. demonstrated that neuronal synchrony in *α*- and *β*-frequencies among frontoparietal and visual regions was strengthened with increasing memory load, and lower WM capacity subjects have lower memory load values which the strength of phase synchrony plateaued (Palva et al. 2010). This result is consistent with our present findings and supports the second hypothesis that the oxy-Hb levels of the lower performer are lower than that of the higher performer even in correct-WM task.

Our evaluation of the two hypotheses to explain how WM performance correlates with NIRS signals leads us to believe that both are correct. Therefore, the correlation between oxy-Hb level and WM performance is caused by differences in activation between correct- and incorrect-WM task performances and the difference in activation for correct-WM tasks between lower and higher performers.

It is known that the oxy-Hb levels in the LPFC with WM can be detected using NIRS (Tsujimoto et al. 2004). However, there is no study that addresses the correlation between NIRS signals and WM performance. In contrast, fMRI studies by Pessoa et al. (2002) show that BOLD signals generated by the LPFC correlate with WM performance. In the EEG/MEG study, Palva et al. (2010) demonstrated that neuronal synchrony predicted the subjects' individual WM capacity. In the EEG study, Vogel and Machizawa (2004) reported that the increase in amplitude of event-related potentials is higher going from two-item trials to four-item trials for subjects with higher WM capacity. Our study seems to be the first report indicating that NIRS signals predict WM performance.

Our results indicate the utility of NIRS for studying cognitive process that require WM. The advantages of using NIRS compared with fMRI are fewer constraints on behavior, inexpensiveness, and the possibility of simultaneous recording with electrical measurements (Huppert et al. 2009; Hoshi et al. 2011; Machado et al. 2011; Tsytsarev et al. 2012). The disadvantages are low spatial resolution and measuring only the surface of the brain (Tsytsarev et al. 2012). In spite of these disadvantages, various studies show NIRS is a really powerful method of functional brain imaging. NIRS in combination with electrophysiological methods was applied to animals performing cognitive task (Fuster et al. 2005). A pain-induced blood flow and an itch-induced blood flow can be separated with NIRS (Lee et al. 2013). Previous study recording with NIRS and fMRI simultaneously in humans shows NIRS signal were significantly correlated with the fMRI signals during WM task (Sato et al. 2013). These previous research shows NIRS appears practical for certain studies of cognitive neuroscience, and we believe our results also supports the utility of NIRS.

Our results facilitate the interpretation of NIRS data acquired from WM experiments. For example, measuring activation with NIRS could mitigate the difficulties associated with intellectual processes that require WM. Furthermore, detecting the fatigue associated with these processes should be possible using NIRS because our results indicate that lower WM performers generate lower NIRS signals. However, our results are derived from the analyses of multiple subjects and not from the changes of WM performance in same subject. Further studies using NIRS to define the relationship between the decline in WM performance and fatigue caused by intellectual work are required.

## Conclusions

We analyzed the correlation between NIRS amplitudes during delay time (keeping the object in mind using WM) and WM performance. Our results showed that subjects with better WM performance had higher oxy-Hb levels compared with control trials with respect to the delay time. These results indicate the possibility of measuring WM performance using NIRS.
